# Robustness of two different methods of monitoring respiratory system compliance during mechanical ventilation

**DOI:** 10.1007/s11517-017-1631-0

**Published:** 2017-02-27

**Authors:** Gaetano Perchiazzi, Christian Rylander, Mariangela Pellegrini, Anders Larsson, Göran Hedenstierna

**Affiliations:** 1 Department of Emergency and Organ Transplant, Bari University, Bari, Italy; 2 Hedenstierna Laboratory, Surgical Sciences, Uppsala University, Akademiska Sjukhuset ing.40 tr.3, 75185 Uppsala, Sweden; 3 Department of Anaesthesia and Intensive Care Medicine, Sahlgrenska University Hospital, Göteborg, Sweden; 4 Hedenstierna Laboratory, Medical Sciences, Uppsala University, Uppsala, Sweden

**Keywords:** Mechanical ventilation, Lung compliance, Neural networks, Acute lung injury, Robustness

## Abstract

Robustness measures the performance of estimation methods when they work under non-ideal conditions. We compared the robustness of artificial neural networks (ANNs) and multilinear fitting (MLF) methods in estimating respiratory system compliance (*C*_RS_) during mechanical ventilation (MV). Twenty-four anaesthetized pigs underwent MV. Airway pressure, flow and volume were recorded at fixed intervals after the induction of acute lung injury. After consecutive mechanical breaths, an inspiratory pause (BIP) was applied in order to calculate C_RS_ using the interrupter technique. From the breath preceding the BIP, ANN and MLF had to compute C_RS_ in the presence of two types of perturbations: transient sensor disconnection (TD) and random noise (RN). Performance of the two methods was assessed according to Bland and Altman. The ANN presented a higher bias and scatter than MLF during the application of RN, except when RN was lower than 2% of peak airway pressure. During TD, MLF algorithm showed a higher bias and scatter than ANN. After the application of RN, ANN and MLF maintain a stable performance, although MLF shows better results. ANNs have a more stable performance and yield a more robust estimation of *C*_RS_ than MLF in conditions of transient sensor disconnection.

**Electronic supplementary material:**

The online version of this article (doi:10.1007/s11517-017-1631-0) contains supplementary material, which is available to authorized users.

## Introduction

Mechanical ventilation is one of the cornerstones of both intensive care therapy and general anesthesia [4, 13]. Increasing complexity in ventilator technology and evidence that mechanical ventilation per se may induce lung injury [29, 36] has prompted the need of improved bed-side monitoring tools. Different methods [10, 16, 19, 38] have been proposed to assess the mechanics of the ventilated lungs during ongoing mechanical ventilation. The most frequently used are algorithms based on multiple linear regression (multilinear fitting, MLF). We have described a system based on artificial neural networks (ANNs) to extract respiratory system compliance (C_RS_) during mechanical ventilation [27] and more recently to assess intrinsic end-expiratory positive pressure (PEEPi) [28].

In this field of research, ANNs have been used during the past years to create intelligent alarms during anesthesia [23], to identify esophageal intubation [22] or to detect lung injury from respiratory tracings [33]. In recent years, ANNs were applied to develop intelligent systems for diagnosing asthma [1], for predicting the outcome of weaning from the ventilator [21], to estimate work of breathing during noninvasive ventilation [2] or to classify lung sounds [25].

Continuous monitoring of vital signs in the clinical setting requires the maintenance of stable performances also in conditions of sensor failure or signal perturbation by noise. This property of estimation methods is defined as *robustness*.

In fact, the progressive developments of monitoring technology will allow the next generation of artificial ventilators to operate also in closed-loop modalities [30]. In this respect, different approaches have been already studied [7], using also the signal of carbon dioxide during anesthesia [8], oxygen [39], electrical diaphragmatic activity [35] or combining different inputs [39]. The necessity of feeding a controller with physiological tracings imposes to develop technologies able to provide robust signals. For this reason, it is important to evaluate conditions that can potentially affect the signals during their daily use.

### Objective

In computer science literature, it is affirmed that both ANN-based [20] and MLF-based [37] methods are inherently *robust*. The aim of the present paper is to evaluate and compare, under the same conditions, the robustness of ANN and MLF in extracting *C*_RS_ when facing signals corrupted by perturbations likely to be found in the clinical environment [6].

## Methods

### Experimental design

We collected tracings of mechanical breaths during controlled mechanical ventilation in a porcine oleic acid model of mild acute respiratory distress syndrome (ARDS) [32].

**Fig. 1:**
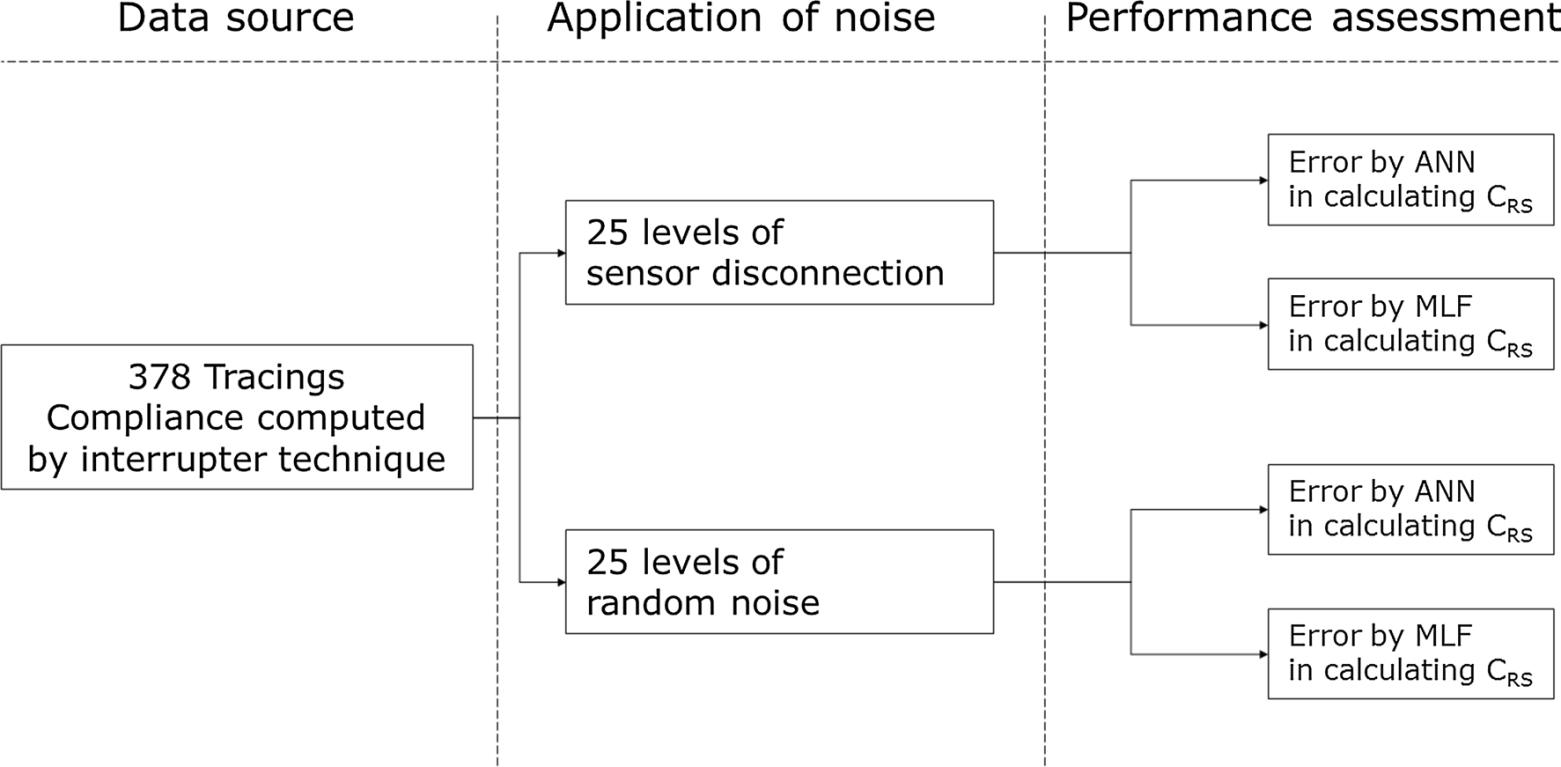
Experimental design. *ANN* artificial neural network, *MLF* multilinear fitting, *C*_RS_ compliance of the respiratory system

Afterward, two different types of signal perturbation were separately applied to the pool of tracings: random noise (RN) and transient disconnection (TD). A previously trained ANN and a MLF algorithm had to extract C_RS_ from these tracings. Robustness of ANN and MLF methods was computed by applying different amplitudes of RN and TD to the pool of MB and measuring the error in estimating *C*_RS_ (see Fig. 1).

### Pool of tracings

A pool of 378 tracings of airway pressure (Paw) and gas flow (V´) was obtained from 24 pigs that had been included in previously published studies [26, 27]. The experimental protocol was approved by the local institutional review board for the care of animal subjects; the care and the handling of the animals were in accordance with the regulations of the Swedish Board for Laboratory Animals and executed following the European Union Directives for animal experiments. After premedication, general anesthesia with muscle relaxation was induced. During the experiment, the main hemodynamic parameters were monitored. The animals were ventilated through a cuffed endotracheal tube by delivering a volume-controlled–constant flow mechanical ventilation (Servo 900 C, Siemens Elema, Solna, Sweden). Tidal volume (*V*_T_) was adjusted to result in normocapnia, and extrinsic positive end-expiratory pressure (PEEPe) was initially set to 5 cmH_2_O. Inspiratory-to-expiratory ratio was set to 1:2 for a respiratory rate of 20 bpm. We induced a mild ARDS lung injury characterized by a PaO_2_/FiO_2_ ratio in the range between 200 and 300 [mmHg] by repeated injections of oleic acid (OA) (Apoteksbolaget, Göteborg, Sweden) into a central vein, targeting a total dose of 0.1 ml/kg. *P*_AW_ and *V′* were measured through a differential pressure transducer (Sensym, SensorTechnics, Pucheim, Germany), connected to the two sampling ports of a D-Lite connector (Datex Ohmeda, Helsinki, Finland) mounted to the endotracheal tube. At the beginning of each experimental session, the transducer was calibrated for pressure and flow measurements using a water column and a precision flow meter (Calibration Analyzer TS4121/P, Timeter Instrument Corporation, St. Louis, MO, USA). Data were sampled at 200 Hz using a LabView-based (National Instruments, Austin, TX, USA) acquisition system. Respiratory tracings were recorded at fixed time intervals: at baseline before OA administration and 5, 20, 35, 50, 65, 95, 125 min after the administration of OA. Each recording session comprised the simultaneous collection of *P*_AW_ and *V′* coming from ten or more consecutive mechanical breaths, followed by a breath with an inspiratory hold maneuver, sustained until a stable pressure plateau had been reached. Two recordings per time interval were performed, with 20 regular breaths in between, allowing the return to a steady-state condition before the second measurement.

Determination of *C*_RS_ was performed by applying the interrupter technique [3] (IT) on the last breath of the sequence, i.e., the one having the end-inspiratory pause, calculating the ratio between *V*_T_/(*P*_plat_-PEEPe), where *P*_plat_ is the plateau pressure. Calculation of *V*_T_ was performed by integrating *V′*.

### Multilinear regression method

This method is based on a first-order mechanical model of the respiratory system. Pressure (*P*_AW,TOT_) in the airways is considered as the sum of elastic (*P*_AW,EL_), resistive (*P*_AW,RES_) and constant components (positive end-expiratory pressure, PEEP):

1 $$ P_{{{\text{AW}},{\text{TOT}}}} = P_{{{\text{AW}},{\text{EL}}}} + P_{{{\text{AW}},{\text{RES}}}} + {\text{PEEP}} $$

MLF stems from the assumption that the elastic and resistive components are linearly related to delivered volume and inspiratory flow, respectively.

2 $$ P_{{{\text{AW}},{\text{EL}}}} = V/C_{\text{RS}} $$

3 $$ P_{{{\text{AW}},{\text{RES}}}} = V^{{\prime }} \times R_{\text{RS}} $$

where *R*_RS_ and *C*_RS_ are the resistance and the compliance of the respiratory system, respectively. Then, Eq. (1) takes the form of

4 $$ P_{{{\text{AW}},{\text{TOT}}}} = V/C_{\text{RS}} + V^{{\prime }} \times R_{\text{RS}} + {\text{PEEP}} $$

Having the tracings of *P*_AW,TOT_, *V* and *V′*, it is possible, by applying the least squares fitting method, to obtain *C*_RS_, *R*_RS_ and PEEP, according to the procedure described by Iotti [16]. In our experiments, the algorithm was implemented on a MATLAB platform (The MathWorks Inc., Natick, MA, USA) and used three simultaneous tracings (*P*_AW,TOT_, *V* and *V′*) in the domain of time.

### Neural network

We trained and validated one ANN to estimate *C*_RS_ during ongoing mechanical ventilation. The method used is described in a paper published by our group [27]. The ANNs used in these experiments were implemented via software on a computer (Neural Networks Toolbox, The MathWorks Inc., Natick, MA, USA). The learning algorithm was resilient backpropagation. The ANNs consisted of three layers, whose activating functions were log-sigmoids for the input and intermediate layer and linear for the output layer. The number of neurons in the input layer was 100 and was determined by the dimensions of the input pattern to be given. The input pattern was the entire inspiratory limb of the volume/pressure loop of the breath to be analyzed. However in order to avoid redundancy of information among neighbor points, the inspiratory limb was under-sampled by taking 50 equally spaced coordinates of pressure and volume. Each curve was rescaled using as scale factor the value of maximum airway pressure (see Fig. 6). After testing for the best architecture, the number of intermediate neurons providing the best performance for the required task was fixed to 25. This was achieved by applying the method of eightfold cross-validation with early stopping [11]. The output layer consisted of 1 neuron, yielding the C_RS_ calculated by the ANN. Training started by dividing the pool of data in two subsets in the ratio of 80:20, using the bigger subset for training and the smaller one to evaluate the learning process. The strategy consisted in training 100 ANNs, all having the architecture described above: the ANN having the lowest mean squared error was the one that we used for the perturbation tests.

### Application of perturbations

#### Random noise

**Fig. 2:**
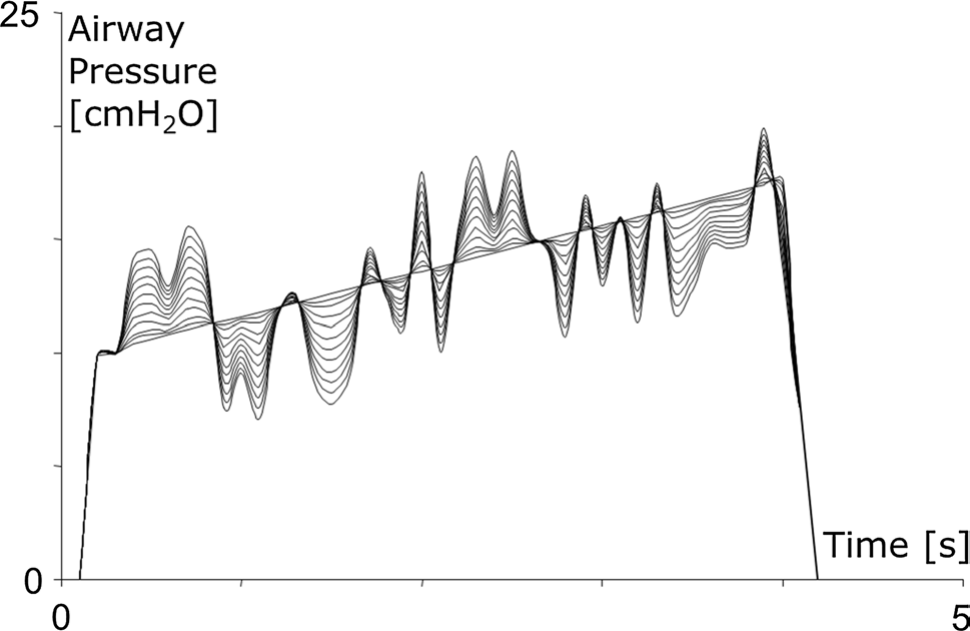
Test of random noise application on inspiratory airway pressure during mechanical ventilation. In this figure, different perturbations are added to the same pressure/time curve, in order to show the effect of different levels of random noise on the same tracing

Random noise (RN) is a non-periodical signal having a flat frequency spectrum [9]. By definition, it has a sampling distribution characterized by having zero mean and defined extrema. In the present paper, the limits of the flat frequency spectrum were defined in proportion to the maximum of the airway pressure (*P*_AW,MAX_) and applied to each pressure tracing (see Fig. 2).

The amplitude of RN varied between 0% of *P*_AW,MAX_ (no RN applied) and 50% of *P*_AW,MAX_ (thus creating a displacement of the original signal that could range between −50 and +50% of *P*_AW,MAX_) in steps of 2%. This way we tested the effects of 25 different levels of RN on the pool of MB. After the application of a given level of RN, MLF and ANN were used to extract C_RS_ from the modified curves. Considering that the true *C*_RS_ had been separately calculated by interrupter technique, it was possible to obtain the mean and the standard deviation of the measurement error according to Bland and Altman [5] on the entire pool of breaths, under the same conditions of noise.

#### Transient disconnection

**Fig. 3:**
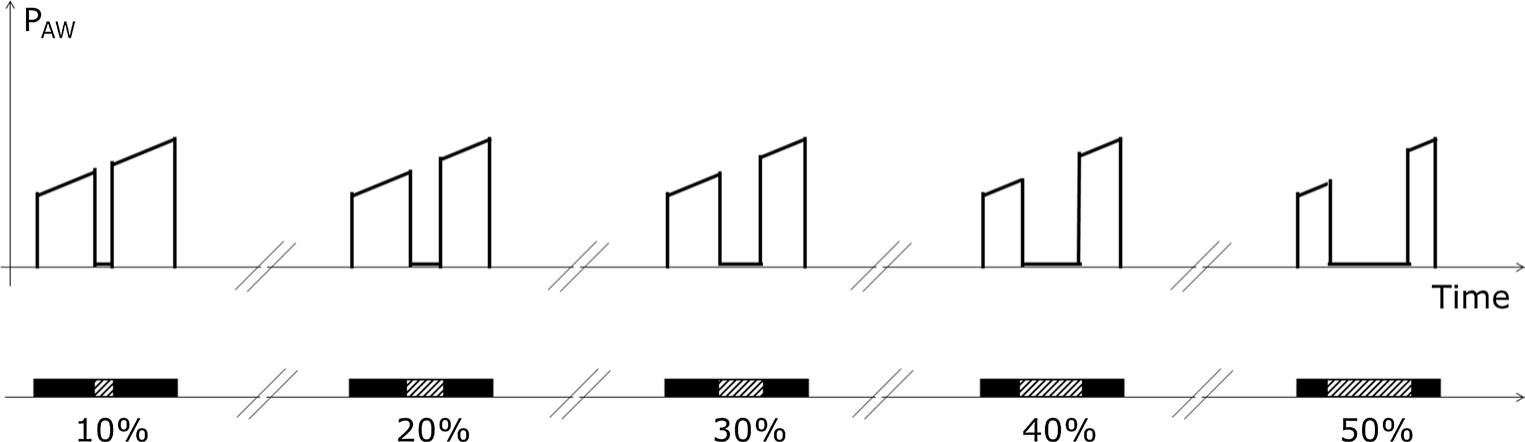
Test of sensor disconnection. Time of sensor disconnection is expressed as percentage of inspiratory time. *P*_AW_ pressure in the airways

During the inspiratory phase of the respiratory cycle, a transient disconnection (TD) of the pressure sensor was simulated. Each level of TD was applied to the entire pool of curves. We imposed TD times lasting from 0 to 50% of the inspiratory time in steps of 2%, thus testing 25 TD levels on the entire pool of MB. Sensor disconnection was generated by zeroing the signal in the central part of the recording (see Fig. 3). As in the case of application of RN, for each level of application of TD on the entire pool of breaths, we calculated the mean and the standard deviation of the measurement error according to Bland & Altman under the same conditions of perturbation.

### Statistics

The estimation error parameters obtained by applying the above-mentioned Bland and Altman approach were subsequently analyzed. Moreover, in order to compare the scatters of the two methods (ANN and MLF) at each level of applied perturbation (RN and TD) separately, the *F* test for variance comparison was performed. The *F* test was preceded by testing that the error measurement population followed a normal distribution. All statistical tests were performed by using the program Minitab ver.14 (Minitab Inc., State College, PA, USA). In all the applied statistical tests, the level of significance *α* was set to 0.01.

## Results

**Fig. 4:**
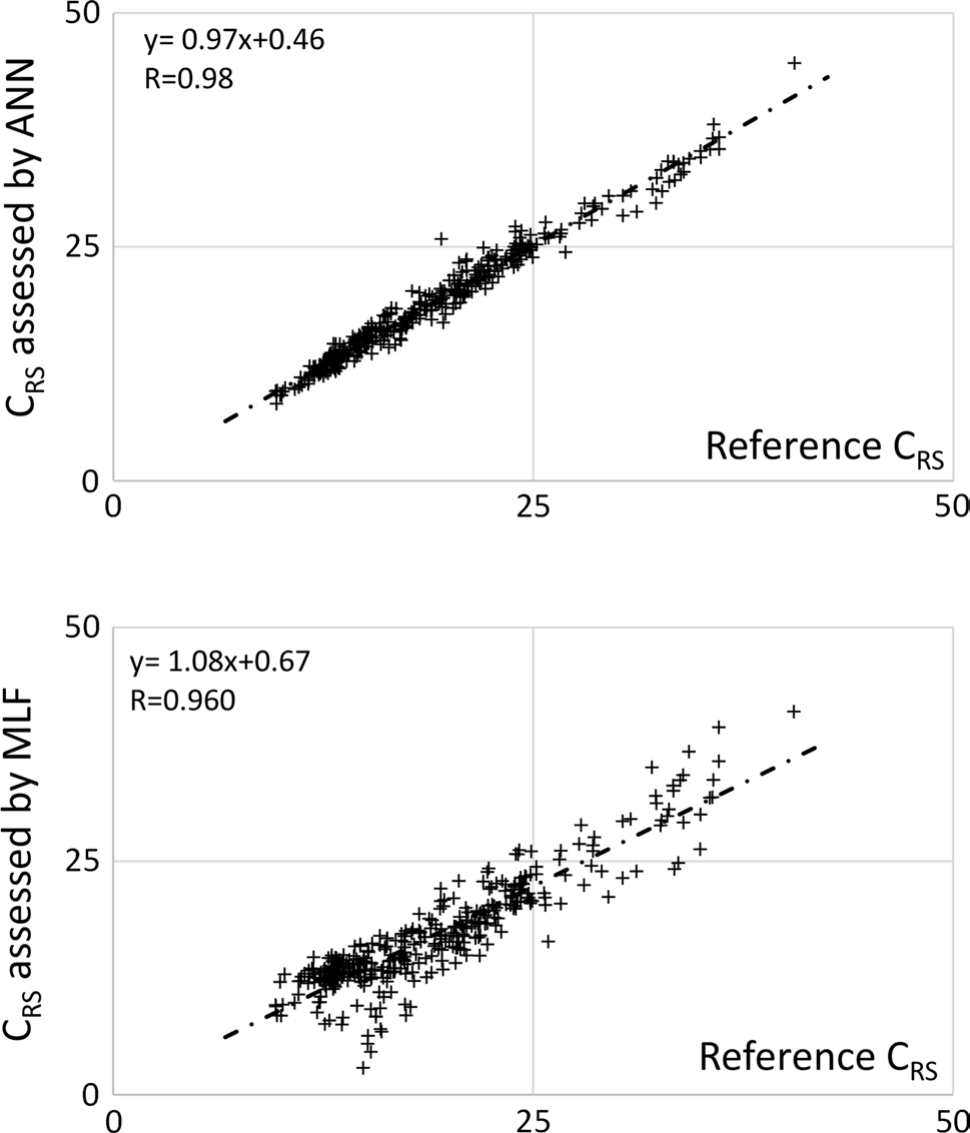
Performance of ANN and MLF in the assessment of the tracings of pressure/volume loop, in the absence of perturbations. In the graphs are reported the parameters of the respective linear regressions

In the absence of perturbations, ANN estimated *C*_RS_ faithfully with negligible bias, presenting a performance (expressed as bias ± standard deviation) of 0.02 ± 1.02 [ml/cmH_2_O]. In the same conditions and using the same notation as above, the MLF algorithm showed a performance of −1.97 ± 2.57 [ml/cmH_2_O]. See Fig. 4.

### Application of random noise (Table 1)

With a perturbation constituted by a RN whose maximum amplitude was 2% of *P*_AW,MAX_, ANN estimated *C*_RS_ with a bias and standard deviation of 1.47 ± 1.87 [ml/cmH_2_O]; with a RN at 50% of *P*_AW,MAX_, performance was expressed by 5.25 ± 5.14 [ml/cmH_2_O].

**Table 1:** Application of random noise

(% of *P*_MAX_)	MLF		ANN	
	Bias	SD	Bias	SD
2	−1.59	2.42	1.47	1.87
4	−1.22	2.29	2.16	3.11
6	−0.86	2.18	2.62	4.08
8	−0.51	2.10	3.02	4.43
10	−0.18	2.04	3.45	4.49
12	0.14	2.00	3.72	4.63
14	0.45	1.98	3.91	4.79
16	0.75	1.98	4.06	4.92
18	1.04	1.99	4.13	5.01
20	1.32	2.01	4.13	5.06
22	1.59	2.05	4.13	5.15
24	1.86	2.10	4.11	5.19
26	2.11	2.15	4.08	5.18
28	2.36	2.21	4.09	5.17
30	2.60	2.27	4.13	5.15
32	2.83	2.34	4.21	5.11
34	3.06	2.41	4.33	5.07
36	3.28	2.48	4.51	5.02
38	3.50	2.56	4.69	4.96
40	3.71	2.63	4.82	4.93
42	3.91	2.71	4.95	4.94
44	4.11	2.78	5.06	4.97
46	4.30	2.86	5.15	5.03
48	4.49	2.93	5.20	5.08
50	4.67	3.00	5.25	5.14

Data are expressed as ml/cmH_2_O

*P*_MAX_ maximum airway pressure, *MLF* multilinear fitting, *ANN* artificial neural network, *SD* standard deviation

MLF showed, in the same conditions, at RN of 2% of *P*_AW,MAX_, a bias and standard deviation of −1.59 ± 2.42 [ml/cmH_2_O]; at RN of 50%, bias and standard deviation were 4.67 ± 3.00 [ml/cmH_2_O] (see Table 1).

**Fig. 5:**
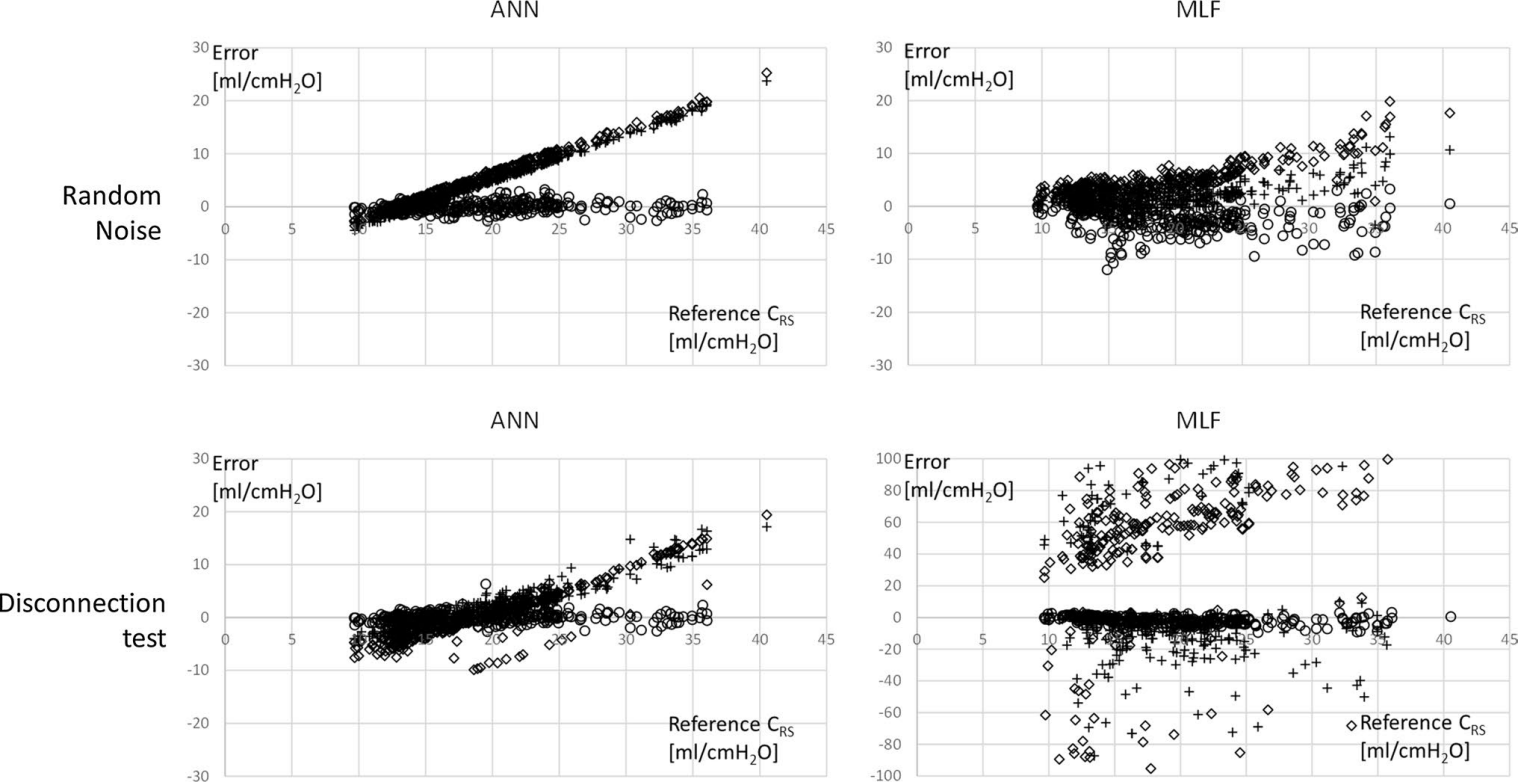
Bland Altman graphs of ANN and MLF performances in facing sensor disconnection and random noise at 0, 24 and 50% of the potential perturbations. On the x-axis is reported the reference measurement; on the y-axis the error in the assessment. Bias and standard deviation were not plotted for sake of clarity. Note the scale of MLF during disconnection test and the fact that in this condition many values can be out of the *graph scale*. *Circles* are measures taken without perturbation; crosses = 24%; squares = 50%. The biases (mean error) and standard deviations are reported in Tables 1 and 2

MLF-based algorithm presented a lower scatter than the ANN-based algorithm throughout the entire test, except when RN was 2%. At each level of applied RN, the population of measurement error was normally distributed. The difference between the two scatters at each level of applied RN was evaluated by the *F* test for variance comparison, and it was found to be statistically significant. See Fig. 5.

### Application of sensor disconnection (Table 2)

When simulating disconnection of the pressure sensor, at 2% of inspiratory time, ANN had a minimal estimation error of 0.13 ± 1.65 [ml/cmH_2_O]. Moreover, the error increased only slightly when the disconnection time increased. Thus, at 50% of inspiratory time, performance was −0.19 ± 4.98 [ml/cmH_2_O].

**Table 2:** Application of sensor disconnection

(% of *T*_I_)	MLF		ANN	
	Bias	SD	Bias	SD
2	−2.95	2.95	0.13	1.65
4	−4.37	4.36	−0.80	2.30
6	−6.46	7.27	0.99	2.96
8	−9.80	13.02	0.95	2.51
10	−16.65	28.66	1.48	3.07
12	−4.72	341.90	2.50	3.16
14	−18.56	352.32	0.82	4.03
16	143.77	2580.33	0.25	3.96
18	−161.26	2176.92	1.52	4.39
20	−27.91	662.73	−0.76	4.32
22	15.30	275.48	0.67	3.95
24	38.44	236.98	1.55	4.02
26	−6.03	359.81	1.52	5.31
28	29.23	226.72	2.04	5.53
30	39.07	328.19	1.28	5.10
32	14.80	159.72	2.17	4.73
34	−59.88	1095.54	2.89	5.29
36	−68.20	1000.96	2.67	5.03
38	−7.03	529.19	−0.71	5.78
40	37.97	505.63	0.97	4.87
42	69.04	429.04	−2.57	6.49
44	43.70	489.92	−1.35	6.50
46	−1101.53	16,718.44	−1.78	6.24
48	5.64	789.16	−3.58	6.56
50	213.71	3918.08	−0.19	4.98

Data are expressed as ml/cmH_2_O

*TI* inspiratory time, *MLF* multilinear fitting, *ANN* artificial neural network, *SD* standard deviation

For the MLF algorithm, at TD equal to 2% of inspiratory time, the error was −2.95 ± 2.95 [ml/cmH_2_O]. The error increased dramatically with increasing disconnection, and at a TD of 50%, it was 213.7 ± 3918 [ml/cmH_2_O]. In estimating C_RS_, ANN showed a lower scatter than MLF under the same conditions of TD.

Analyzing the range of variation of the error parameters, ANN showed a bias (from minimum to maximum) between −3.58 and +2.89 [ml/cmH_2_O]; the bias of MLF method was between −1101.53 and +213.71 [ml/cmH_2_O]. The measurement error was normally distributed at each level of applied TD, for both ANN and MLF. The *F* test for variance comparison revealed that at each level of TD, the scatters of MLF and ANN algorithms were statistically different. See Fig. 5.

## Discussion

**Fig. 6:**
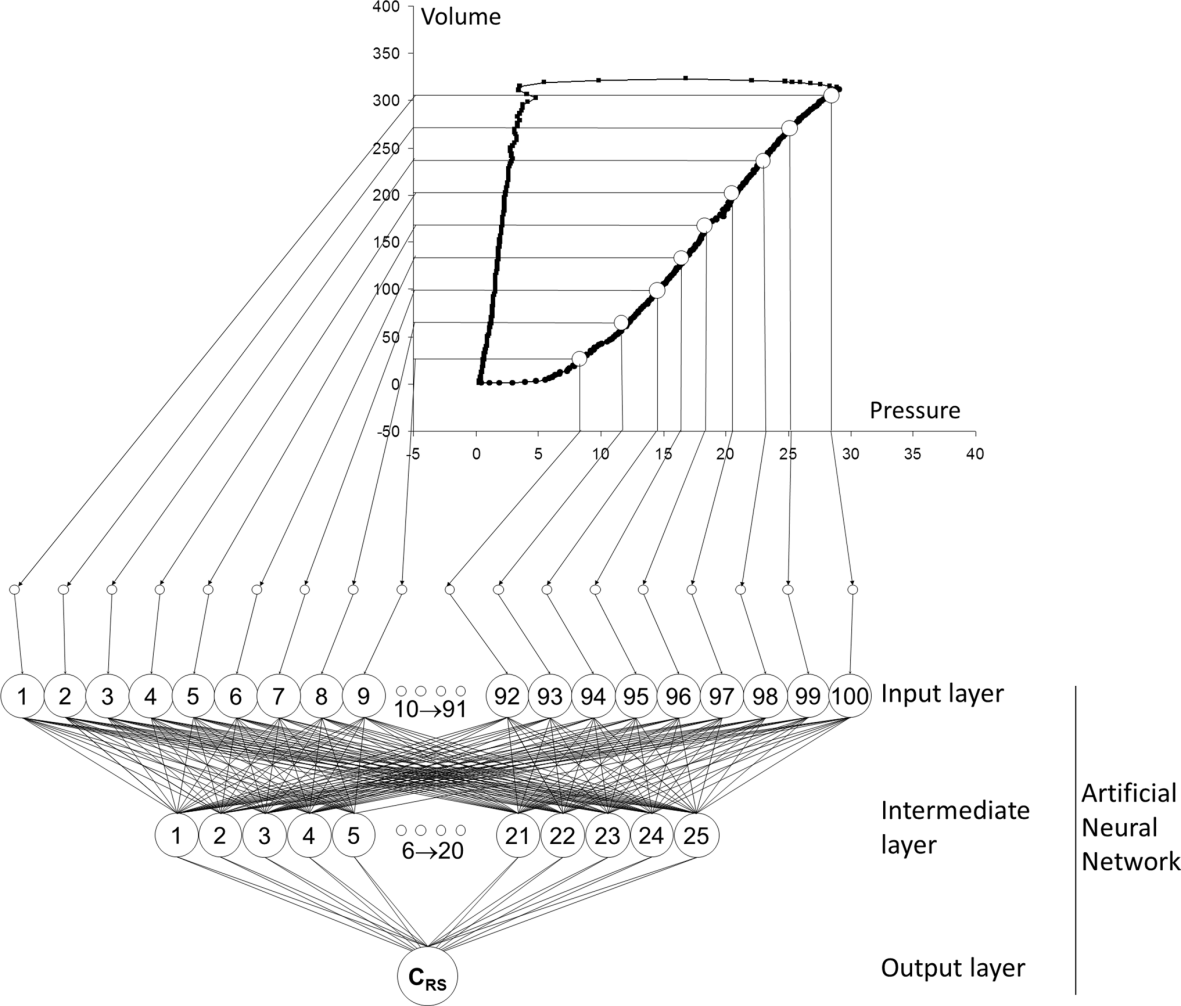
Overview of the method for presenting the pressure/volume curve to the artificial neural network. Fifty iso-spaced pairs of coordinates feed an ANN composed by 100 input neurons, 25 intermediate and 1 output. In order to simulate sensor disconnection, the pressure coordinate of a part of the signal (following the method described in Fig. 3) is switched to zero in the MATLAB script

Robustness of a method is the property for which degradation of performance, in case of corrupted inputs, is slow and smooth. So a method is robust if it works well not only under ideal conditions, but also under conditions representing a departure from an assumed distribution or model [17]. We decided to test random noise and sensor disconnection because they are the two types of perturbation that can be encountered in a clinical environment (Fig. 6).

### About the methods

Random noise represents the extreme condition of all the types of perturbations. It is not periodic and its behavior is unpredictable. It is not deterministic, in the sense that it is not possible to write an equation for predicting its value at any time [9]. In our setting, RN is a model of the interferences arriving on the pressure sensor by other electrical or mechanical devices.

The rationale for implementing tests of disconnection was to study malfunctions depending directly on the pressure sensors. Examples of TD related to mechanical causes can be the transient plugging of the sensor tubing system by water or mucus; other possible correspondence to TD is represented by primary electrical malfunctioning of the pressure transducers.

The family of MLF methods for the extraction of respiratory mechanics comprise algorithms able to decompose a signal into different linear equations in order to obtain information on the properties of the system which generated it. MLF application requires a preconceived idea about the number of variables to be fitted and, more importantly, the assumption that nonlinear components can be considered negligible. This assumption reduces the accuracy in estimating parameters during pathologic conditions, when the respiratory system is no longer behaving as a linear homogeneous system [31]. To overcome these problems, various solutions have been proposed, like the corrections of the algorithm [18] or limiting the application of MLF to specific segments of the breathing cycle [10]. The performance of the MLF algorithm implemented in our paper is in line with previously published literature [16]. One finding in our experiments was a negative bias that was seen even when the signal was uncorrupted. This was not surprising and derives from the fact that MLF methods compute a value of *C*_RS_ that is averaged over the whole breath. It does not take into account the higher compliance that is revealed during a prolonged breath hold. In these conditions, end-inspiratory airway pressure drops, because of stress relaxation or redistribution (“*pendelluft*”) phenomena [24].

Our choice of studying an ANN approach to estimate respiratory mechanics derives from the well-known concept that multilayer perceptrons are universal function approximators [12, 14, 15] irrespective of the degree of nonlinearity. The performance of the ANN implemented in this study, during baseline test conditions (before applying perturbations), is in line with our previous reports [26, 27].

Interrupter technique, being based on static conditions, represents the gold standard for measuring “true” *C*_RS_. In fact, stopping the flow makes it possible to minimize the contribution of resistive components of pressure and of stress relaxation and redistribution phenomena [34].

As suggested by Bland and Altman [5], we analyzed the measurement error in terms of bias and scatter. Bias is the mean difference between the gold standard and the tested method of measurement: it represents the systematic error of the tested method.

The population of measurement errors is distributed around the bias, and its dispersion is estimated by standard deviation. The error scatter around the mean is expression of the precision of the tested method. We wanted to compare precision of the two methods (ANN and MLF) when subjected to the same level of perturbation. In order to obtain this information, we used an *F* test for variance comparison. This test does not take into account the mean of the sample (bias) but only the characteristics of dispersion (scatter). In comparing two measurement methods, it is possible to obtain two similar biases but very different scatters.

We chose *F* test because the question we wanted to address concerned the robustness of the methods when facing perturbations. Evaluation of robustness stems from the analysis of precision (scatter that is measured by variance) at the different level of perturbations.

The ANN used in this experiment was previously trained on the 80% of the 378 tracings and tested on the remaining 20%. At the end of the training, it had a performance expressed by a regression of *y* = 0.97*x* + 0.28 (*R* = 0.98) on the test set of data. Then, the entire pool of 378 curves was used to assess the degradation of its performance when facing sensor disconnection or noise addition. It might be questioned whether the inclusion of examples already “seen” during the training phase could have influenced the results. We used this approach because if we used a testing pool composed only by curves not seen before, we could have not discriminated whether degradation of performance was due to a problem of “teaching strategy” or a problem of not having the capacity of identifying what had been correctly assessed during the training/test. We wanted to analyze separately the degradation of performances from the capacity of the ANN to generalize (i.e., the capacity of applying its knowledge to different conditions), which requires the use of a completely new set of data.

### About results

Our results show that in case of RN application test, the MLF-based method had a lower bias and scatter than the ANN-based algorithm, except for situations in which the application of random noise was lower than 2% of *P*_AW,MAX_. However, the ANN-based algorithm presented a lower bias and scatter than the MLF-based method in the whole TD test.

MLF is slightly or not at all affected by RN application. It maintains its performance throughout the whole test, while the ANN presents a progressively increasing scatter. This “immunity” revealed by MLF on RN is intrinsic to the algorithms itself. The displacement caused by the application of noise has zero mean and so, tracing the curve that has the minimum sum of deviations squared, is equivalent to the original curve. An important difference exists between ANN and MLF in extracting *C*_RS_ when a sensor disconnection occurs. It is possible to observe that ANN during the application of TD continues to yield acceptable solutions, maintaining a good performance in terms of bias and scatter. However, MLF shows extremely high bias and scatter already when 6% of inspiratory time is affected by TD. The sensitivity of MLF also to short transient disconnection may be due to the discontinuity affecting the model to be fitted, no longer interpretable when using a sum of linear equations.

Moreover, while the performance of ANN is expressed by a bias and a scatter included in the range of a few ml/cmH_2_O, in the case of MLF these parameters have a considerably wider range of magnitude. It can be questioned whether so high bias and scatter have any sense in expressing the performance of a measurement system. Reporting these results confirms that MLF algorithm has not a stable performance in case of sensor disconnection.

When designing a monitoring tool, it is possible to apply systems to filter out the perturbations that can affect the measurement system. These systems may preprocess the signal before its arrival to any MLF- or ANN-based modules. However, while periodic noise can be easily filtered, sudden changes in signal characteristics render it unsuitable for feeding a control system. It can be also hypothesized that both methods, ANN and MLF, in relation to their different peculiarities might be used simultaneously in order to ameliorate the global performances of a hypothetical tool applied to the afferent limb of a control system.

Disconnection of signal source could either happen after sensor malfunctioning or endotracheal tube disconnection. This last is a critical event and must be notified by the monitoring tools. Under a clinical perspective, it may be questioned whether a system that tolerates such faults has to be considered secure. However, these two events may be differentiated by monitoring the elapsed time of disconnection: if the time is longer than an acceptable threshold, the alarm for patient disconnection should start.

In principle, an artificial neural network is able to generalize its knowledge to conditions that were not presented during the training phase. However in consideration of its dependency from the choice of the pool of data used for training, we cannot draw any conclusion regarding the applicability of the present ANN to different clinical situations, like different modalities of ventilation or size of the ventilated lungs. Consequently, also the assessment of robustness in such different conditions would require a purposely designed experiment. Our experiments may be classified as a test of the extreme conditions that a signal processing system might undergo during its use in an intensive care setting. Future developments in designing the next generation of mechanical ventilators will have to comply with the increasing need of interfacing different sensors in order to guide ventilators in closed-loop systems [30, 40]. In the field of monitoring and control, robustness is as important as accuracy. ANN-based methods are robust because when they extract information from a curve, they do not require a preconceived model to be fitted. Moreover, the capacity of extracting information from perturbed signals, typical of ANNs, may make them a suitable choice for signal processing, particularly in a “noisy environment” like the intensive care unit.

## Electronic supplementary material

Below is the link to the electronic supplementary material.

Supplementary material 1 (MAT 158 kb)
